# Secreted products of oral bacteria and biofilms impede mineralization of apical papilla stem cells in TLR-, species-, and culture-dependent fashion

**DOI:** 10.1038/s41598-018-30658-5

**Published:** 2018-08-21

**Authors:** Xenos Petridis, Luc W. M. van der Sluis, René J. B. Dijkstra, Marja G. L. Brinker, Henny C. van der Mei, Martin C. Harmsen

**Affiliations:** 1 University of Groningen, University Medical Center Groningen, Center for Dentistry and Oral Hygiene, Groningen, The Netherlands; 2 University of Groningen, University Medical Center Groningen, Department of Pathology and Medical Biology, Groningen, The Netherlands; 3 University of Groningen, University Medical Center Groningen, Department of Biomedical Engineering, Groningen, The Netherlands

## Abstract

Regenerative endodontics exploits the mineralization potential of stem cells from the apical papilla (SCAPs) in order to promote root maturation of permanent immature teeth. SCAPs may encounter post-disinfection residual bacteria either in planktonic or in biofilm growth mode. Bacterial components bind to Toll-like receptors (TLRs) and trigger pro-inflammatory responses. We hypothesized that biofilm-triggered TLR activation affects the mineralization potential of human SCAPs. SCAPs were challenged with conditioned media derived from standardized dual-species biofilms and planktonic bacterial cultures and their inflammatory status and mineralization capacity were studied. Bacterial products from both growth modes (planktonic *vs*. biofilm) compromised cell viability, proliferation and mineralization capacity of SCAPs, but in a species- and growth mode-dependent fashion. While *TLR4* expression remained unaffected, *TLR2* expression was upregulated coinciding with a pro-inflammatory activation of SCAPs. Moreover, TLR and its downstream TGF-β-associated kinase (TAK1) appeared to be blocking mineralization, as inhibition of these factors restored it. In conclusion, bacterial products promoted the pro-inflammatory status and inhibited mineralization of human SCAPs in a TLR-, species-, and culture-dependent fashion. TLR2 emerged as the pivotal mediator of these responses and further research is warranted towards the judicious manipulation of SCAPs in order to modify the untoward events of TLR-priming and signaling.

## Introduction

Dental caries and traumatic injuries constitute the major risk factors for the development of endodontic disease. These conditions create an adverse microenvironment, in which the dental pulp is compromised by invading opportunistic bacterial pathogens of the oral flora. Without treatment, inevitably pulpal necrosis will occur together with root canal system infection (endodontic infection) and inflammation that causes bone resorption (apical periodontitis). In adolescents, where root development is still in progress, these infections inhibit further root formation, which reduces the long-term tooth survival. Current regenerative endodontic procedures include the disinfection of the root canal system that generates a conductive environment to heal the apical periodontitis and that is inductive to deposit mineralized tissue and complete the root maturation^1^.

The current clinical procedures start with disinfection regimens^2^, which facilitate further root maturation by local stem cells. These stem cells reside mainly in the most apical part of the dental papilla, namely the apical papilla (stem cells from the apical papilla or SCAPs) and exhibit a high mineralization potential^3,4^. Unfortunately, it proves impossible to fully sterilize an infected root canal environment in teeth with incomplete root development^5^. This is partly due to the minimal mechanical preparation and cautious irrigation that are employed to prevent loss/washing out of important repair cells, such as SCAPs^6,7^. Therefore, while dental pulp regeneration after these disinfection procedures is sub-optimal at best^8–11^, it is on the other hand not compromised in the absence of bacteria^12–18^. Interestingly, residual infections negatively impact on the mineralization process of root formation in immature teeth^19^.

Pathogen associated molecular patterns (PAMPs), such as lipopolysaccharides (LPS) or whole bacterial extracts, inhibit the mineralization capacity of human dental pulp stem cells (DPSCs)^20,21^. In fact, also a mono-species biofilm disrupted the mineralizing capacity of SCAPs *in vitro*^22^. This is relevant because biofilms occur naturally in the root canal system^23^ and produce PAMPs that might affect the phenotype of residing cells^24^.

Bacterial components act on cells via binding and activation of specific receptors including the Toll-like receptors (TLR)^25^. TLR expression and signaling is of particular importance on phagocytic immune cells, but expression of TLRs is ubiquitous and includes mesenchymal stem cells (MSCs) as well^26–29^. However, the downstream influence of TLR activation on the mineralization potential of MSCs is variable and depends on the type of agonist, the type of TLR and the origin of the MSCs^26,27,30–33^. In particular, the expression of TLR2 by human DPSCs^34^ seems to be relevant for endodontic regenerative procedures as, *in vitro*, LPS activates TLR2 on DPSCs causing an inhibition of mineralization capacity^20^. In fact, Gram-positive bacteria show a strong persistence and may survive root canal disinfection procedures^35,36^, after which these interact with homed SCAPs. This interaction is dictated by structural components, such as lipoproteins, lipoteichoic acid (LTA) and peptidoglycans which constitute the major TLR2-inductive PAMPs^37^. The further development and optimization of endodontic regenerative procedures would benefit from a better understanding of the consequences of TRL2 activation in SCAPs.

Regenerative endodontic procedures in infected immature teeth result in an encounter between homed SCAPs and bacterial/biofilm components. To establish predictable clinical protocols promoting intra-radicular hard tissue formation, the influence of bacteria/biofilm on SCAP mineralization efficiency and the associated signaling pathways are important. Here we investigated the response of SCAPs, in terms of basic cellular function and mineral formation, when exposed to bacterial and biofilm secreted products and whether TLR2 signaling plays a role in the mineralizing fate of bacterial/biofilm-primed SCAPs.

## Methods

### Isolation, culture and characterization of human SCAPs

The use of extracted teeth was approved for research purposes by the Institutional Review Board of the University Medical Center Groningen (Medisch Ethisch Toetsingscommissie, Universitair Medisch Centrum Groningen, registration number 201501165). The donors did not participate in any other part of the experimental protocol. Therefore, the study was judged as not falling under the scope of the Medical-Scientific Act for research with humans (METc 2015.584). Collection of extraction teeth was performed in accordance with the relevant guidelines and regulations after informed consent was obtained from the participants. The apical papilla was retrieved from immature impacted third molars that were extracted from young patients (age 16–18 years) who presented for scheduled tooth extraction at the Oral and Maxillofacial Surgery Department, University Medical Center Groningen, the Netherlands, for reasons not related to our experimental protocol.

Immediately after extraction, the teeth were placed in ice-cold DMEM (Lonza Biowhittaker, Verviers, Belgium), supplemented with 2 mM L-glutamine (Lonza Biowhittaker, Verviers, Belgium), 300 U/mL Penicillin/Streptomycin (Gibco, Invitrogen, Carlsbad, CA) and 0.75 μg/mL amphotericin B (Fungizone®, Gibco, Invitrogen). The apical papilla was separated from the root tips with forceps and minced into tiny pieces with a scalpel. The minced pieces were digested in PBS containing 3 mg/mL collagenase type I (Sigma Aldrich), 4 mg/mL dispase (Sigma Aldrich), 300 U/mL Penicillin/Streptomycin and 0.75 μg/mL amphotericin B under constant agitation, at 37 °C for 1 h. After digestion, 5 volumes of DMEM, supplemented with 10% foetal bovine serum (FBS) (Thermo Scientific, Hemel Hempstead, UK), 200 U/mL Penicillin/Streptomycin, 0.50 μg/mL amphotericin B and 2 mM L-glutamine was added and centrifuged (300 × g, 4 °C, 5 min). Cells were re-suspended in the above culture medium and passed through a 70-μm strainer (Beldico, the Netherlands) to obtain single-cell suspensions. The cells were seeded in 25-cm^2^ culture flasks (Corning® Costar®, Sigma-Aldrich) and incubated in a humidified incubator at 37 °C with 5% CO_2_. Media were refreshed after 3 days. After 1 week, DMEM supplemented with 10% FBS, 2 mM L-glutamine and 100 U/mL Penicillin/Streptomycin was added (hereafter called culture medium). After reaching 80–90% confluency, cells were transferred in a 75-cm^2^ culture flask (Corning® Costar®, Sigma-Aldrich) (passage 1) and thereafter sub-cultured at a ratio 1:3. SCAPs from passages 3–5 were used for all experiments.

To determine CD surface marker expression, SCAPs from passage 3 were analysed by flow cytometry (FACS) (FACSCalibur, BD Biosciences, Franklin Lakes, NJ, USA). The following fluorochrome-conjugated anti-human monoclonal antibodies were used: CD31-phycoerythrine/cyanine7 (Pe/Cy7; IQ Products, Groningen, The Netherlands), CD45-fluorescein isothiocyanate (FITC; IQ Products), CD44-FITC (BD Bioscience), CD29-APC (eBiosience, Vienna, Austria), CD90-allophycocyanin (APC; BD Bioscience, San Jose, CA, USA) and CD105-Pe/Cy7 (eBioscience). Details are provided as Supplementary Methods S1. To confirm their multilineage differentiation potential, SCAPs from passage 3 were assessed for their osteogenic, adipogenic and smooth muscle differentiation capacity. Details are provided as Supplementary Methods S2.

### Bacterial Strains and Growth Conditions

The clinical isolates *Streptococcus oralis* J22 and *Actinomyces naeslundii* T14V-J1 were used. The bacteria were streaked on blood agar plates and grown aerobically for *S. oralis* and anaerobically for *A. naeslundii* at 37 °C for 24 h. A single colony was used to inoculate 10 mL modified Brain Heart Infusion broth (37.0 g/L BHI, 1.0 g/L yeast extract, 0.02 g/L NaOH, 0.001 g/L Vitamin K1, 5 mg/L L-cysteine-HCl, pH 7.3) (BHI, Oxoid Ltd., Basingstoke, Hampshire, UK) and both bacteria were grown for 24 h under the appropriate conditions (pre-cultures).

Pre-cultures were used to inoculate 50 mL modified BHI (1:20 dilution) and grown for 16 h (main cultures). Bacteria were harvested by centrifugation (6350 × g, 10 °C, 5 min) and washed twice in sterile adhesion buffer (0.147 g/L CaCl_2_, 0.174 g/L K_2_HPO_4_, 0.136 g/L KH_2_PO_4_, 3.728 g/L KCl, pH 6.8). The bacterial pellets were suspended in 10 mL sterile adhesion buffer and sonicated intermittently in ice-water for 3 × 10 sec at 30 W (Vibra cell model 375, Sonics and Materials Inc., Newtown, CT, USA) to break bacterial chains or aggregates. Bacteria were counted in a Bürker-Türk counting chamber (Marienfeld-Superior, Germany) and diluted in sterile adhesion buffer.

### Biofilm Formation in Constant Depth Film Fermenter (CDFF)

The CDFF^38^ was equipped with 15 sample holders and each sample holder with 5 saliva-coated hydroxyapatite (HA) discs, recessed to a depth of 250 µm. For the saliva coating of the HA disks, freeze-dried whole saliva from at least 20 healthy volunteers of both genders was used^39^. All volunteers gave their informed consent to saliva donation, in agreement with the guidelines set out by Institutional Review Board of the University Medical Center Groningen, the Netherlands. Briefly, the freeze-dried saliva was dissolved in 30 mL adhesion buffer (1.5 g/L), stirred for 2 h and centrifuged at 10,000 × g, 10 °C for 5 min. Then, the HA discs used for biofilm growth were placed in the sample holders and exposed to the reconstituted saliva under static conditions, at 4 °C for 14 h. Subsequently, the sample holders were rinsed with adhesion buffer, placed in the sterile CDFF and 200 mL of a dual-species bacterial suspension consisting of 6 × 10^8^ bacteria/mL for *S. oralis* J22, and 2 × 10^8^ bacteria/mL for *A*. *naeslundii* T14V-J1 was introduced in the CDFF. The bacterial suspension was admitted under continuous supply (flow rate 100 mL/h), while the CDFF table with the sample holders was rotating slowly (1 rpm). After 2 h, the rotation was stopped for 1 h to allow for bacterial adhesion onto the saliva-coated HA discs. Finally, rotation was resumed (8 rpm) and biofilms were grown under continuous supply of modified BHI (flow rate of 45 mL/h), at 37 °C, for 96 h in the CDFF.

### Bacterial conditioned media

After 96 h of incubation in the CDFF, 10 biofilm-carrying HA disks were first dipped three times in sterile PBS (to remove non-adherent bacteria), then transferred into 50 mL antibiotic-free cell culture medium (DMEM supplemented with 10% FBS and 2mM L-glutamine) in sterile beakers and incubated in ambient air, at 37 °C for 16 h. Next, the biofilm conditioned medium with the biofilm-carrying HA discs were sonicated in iced water, intermittently, at 30 W, for 2 min. Intermittent sonication in iced water, at 65 W, for 2 min followed for further mechanical disruption of biofilms and release of tightly bound extracellular polymeric substances (EPS) components from the biofilm matrix in the conditioned medium^40^. Collection of the conditioned media containing soluble extracellular products secreted from the organized bacterial communities as well as loosely and tightly bound EPS from the biofilm matrix was done by centrifugation (10,000 × g, 10 °C, for 5 min). Finally, the supernatant was passed through a 0.2 μm filter (Corning). Sterility of the conditioned medium was confirmed by spot plating 10 µL aliquots on blood agar plates followed by overnight incubation in aerobic and anaerobic conditions.

For the collection of conditioned media from the mono-species grown in planktonic cultures of, *S. oralis* J22 and *A*. *naeslundii* T14V-J1 derived from the respective main cultures were washed twice with PBS, centrifuged (6350 × g, 10 °C, 5 min) and suspended in 10 mL PBS. Next, bacteria were counted and 1 × 10^8^ bacteria/mL suspended in 50 mL antibiotic-free culture medium (DMEM supplemented with 10% FBS and 2mM L-glutamine). The planktonic cultures were incubated in ambient air, at 37 °C for 16 h for *S. oralis* and in an anaerobic chamber, at 37 °C for 16 h for *A. naeslundii*. The conditioned media containing bacterial components and soluble secreted extracellular products were collected by centrifugation (10,000 × g, 10 °C, for 5 min), after an intermittent sonication in iced water, at 30 W, for 2 min. Following, the supernatants were filtered through a 0.2 μm filter. Sterility was confirmed by spot plating 10 µL aliquots on blood agar plates followed by overnight incubation in the appropriate conditions.

For the collection of conditioned media from the dual-species grown in planktonic culture the mono-species conditioned were combined. Briefly, due to the predominance of *S. oralis* J22 in the cultures and the lack of resemblance to the bacterial composition encountered in the CDFF dual-species biofilms (3:1 ratio for *S. oralis* J22 and *A. naeslundii* T14V-J1 respectively), collected conditioned media from the mono-species grown in planktonic cultures were combined in a ratio 3 (*S. oralis*):1 (*A. naeslundii*).

### Optimization of conditioned media dilution

In order to find the maximum concentration of conditioned media capable of inducing an effect and maintaining the SCAPs population for extended time periods, a serial dilution cytotoxicity assay based on the conversion of the methyl-thiazolyl-diphenyl tetrazolium bromide (MTT) was performed. Details are provided as Supplementary Methods S3.

### MTT assay (for assessing cell viability as a surrogate outcome)

Human SCAPs (passage 3) were seeded onto flat bottom 96-multiwell plates at a cell density of 1 × 10^3^ cells/well and incubated with 150 µL culture medium in a humidified incubator at 37 °C with 5% CO_2_ for 24 h. Next, the spent media were decanted and 150 µL of the different conditioned media was added to each well. Cells cultured in culture media served as controls. The media were refreshed every 48 h. At the end-point of the assay (7 days), the wells were washed once with 150 µL PBS and 150 µL of the different conditioned media (or culture media for the control group) containing 0.5 mg/mL MTT was added. The plates were incubated for 4 h in a humidified incubator at 37 °C with 5% CO_2_. Following incubation, media were decanted, 150 µL of DMSO (Dimethyl Sulfoxide) (Sigma-Aldrich, Amsterdam, the Netherlands) was added to each well and the plates were covered with aluminium foil and agitated on an orbital shaker for 15 min. Next, the absorbance was measured at a wavelength of 570 nm (with a reference filter of 650 nm) with a Benchmark microplate reader (Bio-Rad Laboratories, Hercules, CA). The assay was repeated 3 independent times (cell cultures) with triplicate samples for each group.

### Proliferation assay

Human SCAPs (passage 3) were seeded onto flat bottom 96-multiwell plates at a cell density of 1 × 10^3^ cells/well and grown as in the former section. After 7 days, immunocytochemical staining of the human Ki-67 proliferation marker was performed. Details of the staining protocol are provided as Supplementary Methods S4. The TissueFAXS microscopy system (TissueGnostics GmbH, Vienna, Austria) was used to fully scan each well with the DAPI and Texas red filters at 10x magnification sequentially. Analysis of the captured images was carried out with Tissue Quest 4.01.0127 software (TissueGnostics GmbH, Vienna, Austria). Results were expressed as % of positive cells for Ki-67 to the total of DAPI stained cells. The assay was repeated 3 independent times (cell cultures) with triplicate samples for each group.

### Mineralization

Human SCAPs (passage 3) were seeded onto 12-well plates at a cell density of 4 × 10^4^ cells/well and incubated with culture medium. Mineralizing conditioned media (mDMEM) were prepared by first diluting the different conditioned media in culture media to their optimum maximum concentration and then by adding 10^−8^ M dexamethasone, 5 mM β-glycerophosphate, 50 μM ascorbic acid and 2 mM KH_2_PO4 (all reagents from Sigma, St Louis, MO, USA). Upon reaching confluency, the spent media were decanted and 1 mL from the different mineralizing conditioned media was added. Cells were cultured for 14 days with media refreshment every 3 days. Subsequently, cells were fixed with 2% PFA in PBS for 30 min, washed three times with PBS and incubated with PBS containing 1 µg/ml 4′,6-diamidino-2-phenylindole (DAPI), in room temperature and dark conditions, for 15 min. The TissueFAXS microscope system was used to fully scan each well with the DAPI filter at 10x magnification and quantification of the DAPI stained nuclei was carried out with Tissue Quest 4.01.0127 software. Next, the wells were washed three times with sterile demineralized water and incubated with 40 mM Alizarin Red-S (AR-S) (pH 4.2), in room temperature under gentle shaking for 20 min. The non-incorporated dye was aspirated and the wells were washed five times with sterile demineralized water for reduction of the unspecific staining. Finally, the wells were scanned again using the TissueFAXS microscope system and images were captured before applying an established protocol for extraction and quantification of AR-S from the stained monolayers^41^. Details of the protocol for dye extraction and quantification are provided as Supplementary Methods S5. The moles of calcium deposited per cell were extrapolated based on the principle that 1 mole of AR-S binds to 2 moles of calcium^42^. The staining was repeated 3 independent times (cell cultures) with triplicate samples for each group and during quantification triplicate absorbance read-outs were performed.

### Gene expression analysis

Human SCAPs (passage 3) were seeded onto 6-well plates at a cell density of 1 × 10^5^ cells/well and incubated with culture medium. Upon reaching confluency, the spent media were decanted and 2 ml from the different mineralizing conditioned media was added. The samples were cultured for 14 days with media refreshment every 3 days. Total RNA was extracted with the RNeasy® Plus Mini Kit (Qiagen), according to manufacturer’s instructions. Concentration and purity of the extracted RNA were checked by means of a spectrophotometer (NanoDrop 2000, Thermo Scientific, Pittsburgh, PA). Details about the complementary DNA synthesis are provided as Supplementary Methods S6.

Gene expression levels were determined by RT-qPCR for *DSPP*, *ALPL*, *BGLAP*, *BMP2*, *BMP7*, as mineralization differentiation marker genes, *TNFA*, *IL6*, *IL8* as inflammation marker genes and *TLR2* and *TLR4*, as immunophenotypical transition marker genes. All primers were purchased from Sigma-Aldrich (KiCqStart™ Primers) and are listed in Supplementary Table 1. The detailed RT-qPCR protocol is provided as Supplementary Methods S7. The expression levels of the target genes were obtained via the ΔΔC_T_ method after normalization using β2 microglobulin (*B2M*) as reference gene and as relative induction compared to non-mineralized control. The assay was repeated three times independently (cell cultures) with duplicate samples.

### TLR2/4 blocking and mineralization

TLR2/4 signaling was inhibited with OxPAPC (InvivoGen, San Diego, CA, USA), a mixture of oxidized phospholipids competing upstream with the TLR2/4 accessory proteins. Additionally, in a separate set of experiments, a pivotal downstream mediator of TLR2/4 signaling, TGF-β-associated kinase – TAK1, was also inhibited with 5Z-7-oxozeaenol (Sigma-Aldrich). Human SCAPs (passage 3) were seeded onto 48-well plates at a cell density of 2 × 10^4^ cells/well and incubated with culture medium. Upon reaching confluency, the spent media were decanted and 0.5 mL from the different mineralizing conditioned media containing either 5 μM OxPAPC (reconstituted in chloroform) or 6 μM 5Z-7-oxozeaenol (reconstituted in DMSO) were added. The cells were cultured for 14 days, with the mineralizing conditioned media and the inhibitors being refreshed every 3 days. Cells incubated with the different mineralizing conditioned media containing chloroform and DMSO, served as controls. Following, an *in vitro* functional mineralization assay based on AR-S staining and quantification was performed as described above.

### Statistical analysis

Statistical analysis was performed using SPSS 22.00 software (SPSS Inc., USA). All data were expressed as Mean ± Standard Deviation (SD). Normality of data distribution was assessed with the Shapiro-Wilk test. One-way analysis of variance (ANOVA) with a Tukey’s Honest Significant Difference (Tukey’s HSD) multiple comparison *post-hoc* test was performed to assess SCAPs cell viability, proliferation capacity, mineralization efficiency and relative gene expression (log_2_ mRNA fold-change) after exposure to the various conditioned media (a = 0.05). Independent unpaired Student’s t-test was used to compare differences in the mineralizing efficiency between each pair of similarly exposed SCAPs, with and without the addition of the TLR2 inhibitors (a = 0.05).

## Results

### Morphological characteristics, immunophenotypical analysis of cell-surface markers and multilineage differentiation capacity of isolated human SCAPs

Confluent cultures of human SCAPs at passage 3 exhibited fibroblast-like morphological characteristics, with elongated and spindle-shaped cells present in the cell culture. The multilineage differentiation of SCAPs towards osteogenic, adipogenic and smooth-muscle was verified by AR-S, Oil-Red-O and phalloidin-FITC staining respectively. Furthermore, immunophenotypical analysis of CD surface markers, revealed high positivity for markers associated with mesenchymal stromal cell phenotypes (CD- 44, 29, 90, 105) and extremely low positivity for markers associated with endothelial (CD31) and hematopoietic (CD45) cells (Supplementary Fig. S1).

### Determination of the optimal maximum concentration of conditioned media

The initial MTT-based cytotoxicity screening of the different conditioned media yielded a concentration of 25% conditioned media as the optimal maximum concentration of exposure. SCAPs mitochondrial activity seemed to be unaffected by any concentration changes until the 25% concentration cut-off point, with any increase further causing an abrupt decline on cells’ viability. The same dose-response curve applied for all different conditioned media (Supplementary Fig. S2).

### SCAPs cell viability is diversely affected, depending on the imposed stimuli

**Figure 1:**
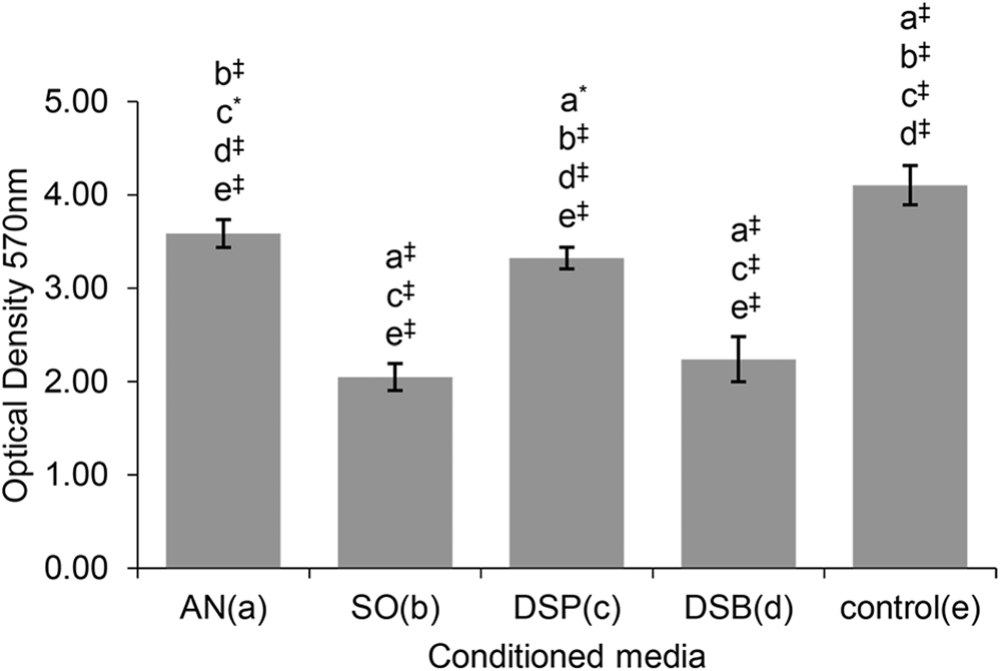
Comparison of cell viability of human SCAPs after 7-day exposure to the different conditioned media (one-way *ANOVA* test, [F(4,40) = 222.042, P < 0.001]). Bars represent mean values; error bars indicate Standard Deviation (SD). Each small letter in parentheses (a–e) represents a respective group of conditioned media. The small letters above the error bars of each group indicate significant differences with the other groups, as yielded by the multiple comparisons test (*post-hoc Tukey HSD* test). Statistical significance is represented by * for P ≤ 0.05 and ^‡^ for P ≤ 0.001. AN, *A. naeslundii* T14V-J1; SO, *S. oralis* J22; DSP, dual-species planktonic; DSB, dual-species biofilm; control, culture medium (DMEM).

Irrespective of the origin of the conditioned media, these all significantly reduced the cell viability of SCAPs compared to non-stimulated controls (DMEM), as assessed by MTT conversion (P ≤ 0.001, Fig. 1). Conditioned media from planktonic *S. oralis* J22 planktonic and dual species biofilm cultures most strongly suppressed cell viability, followed by the dual species planktonic and *A. naeslundii* T14V-J1 cultures (Supplementary Tables 2–4).

### SCAPs proliferation is reduced in a bacterial species-dependent fashion

**Figure 2:**
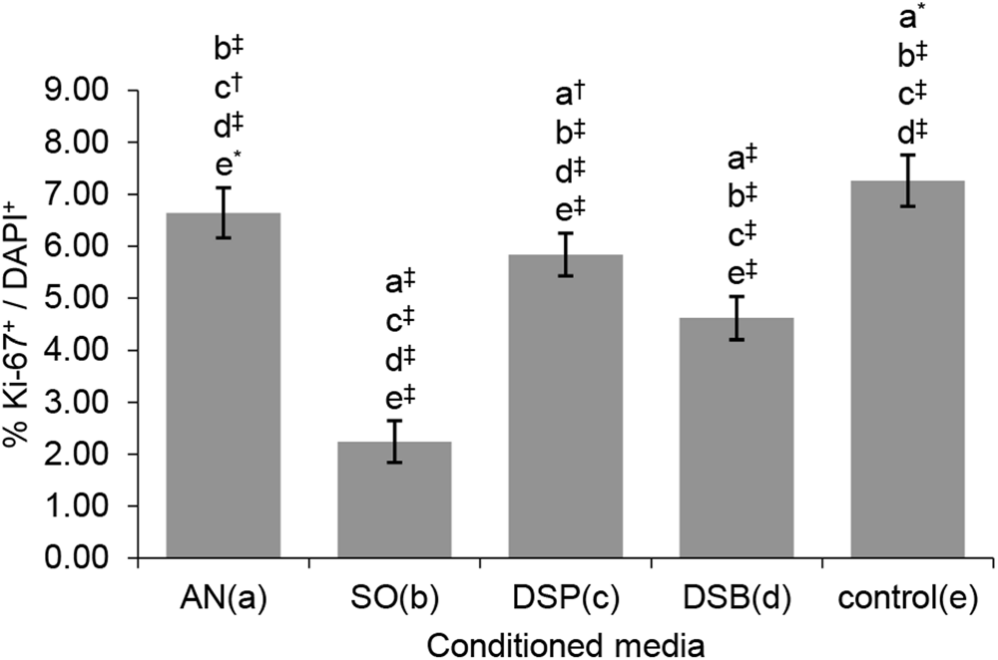
Comparison of the proliferation capacity of human SCAPs after 3-day exposure to the different conditioned media (one-way *ANOVA* test, [F(4,40) = 181.487, P < 0.001]). Bars represent mean values; error bars indicate Standard Deviation (SD). Each small letter in parentheses (a–e) represents a respective group of conditioned media. The small letters above the error bars of each group indicate significant differences with the other groups, as yielded by the multiple comparisons test (*post-hoc Tukey HSD* test). Statistical significance is represented by * for P ≤ 0.05, ^†^ for P ≤ 0.01 and ^‡^ for P ≤ 0.001. Ki-67+, Ki-67 positive SCAPs; DAPI+, DAPI positive SCAPs; AN, *A. naeslundii* T14V-J1; SO, *S. oralis* J22; DSP, dual-species planktonic; DSB, dual-species biofilm; control, culture medium (DMEM).

Irrespective of the origin of the conditioned media, these all significantly reduced the proliferation of SCAPs compared to non-stimulated controls (DMEM), as assessed by the immunocytochemical Ki-67 nuclear staining (P ≤ 0.001, except for *A. naeslundii* T14V-J1 where P ≤ 0.05). Conditioned media from *S. oralis* J22 planktonic and dual species biofilm cultures most strongly inhibited SCAPs proliferation, followed by the dual species planktonic and *A. naeslundii* T14V-J1 cultures (Fig. 2) (Supplementary Tables 5–7).

### Mineralization of SCAPs is inhibited in a bacterial species and culture-dependent fashion

**Figure 3:**
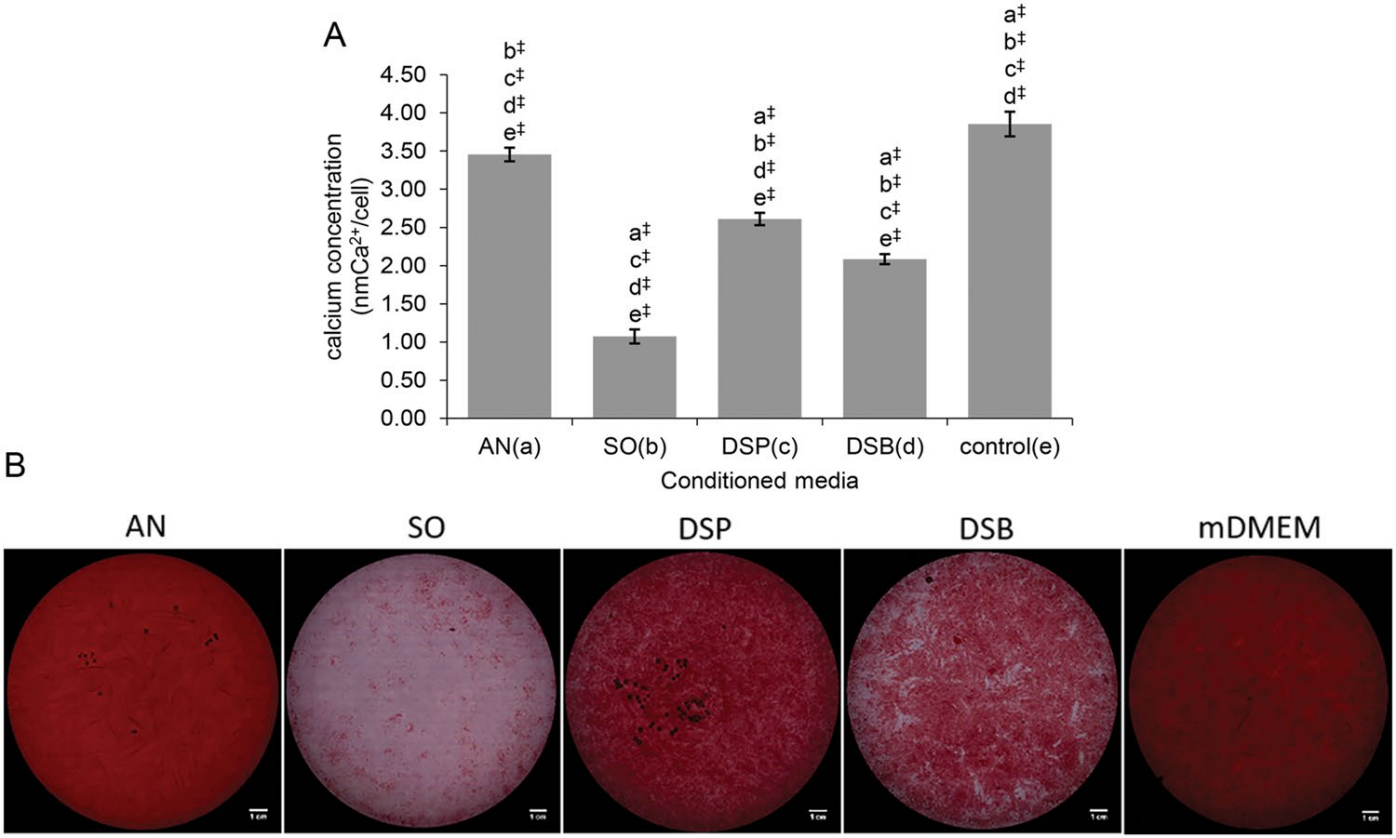
Mineralization capacity of human SCAPs exposed to the different mineralizing conditioned media. **(A)** Comparison of the extracellular calcium deposited by human SCAPs (normalized per cell level) after 14-day exposure to the different mineralizing conditioned media (one-way *ANOVA* test, [F(4,40) = 1000.310, P < 0.001]). Bars represent mean values; error bars indicate Standard Deviation (SD). Each small letter in parentheses (a–e) represents a respective group of conditioned media. The small letters above the error bars of each group indicate significant differences with the other groups, as yielded by the multiple comparisons test (*post-hoc Tukey HSD* test). Statistical significance is represented by ^‡^ for P ≤ 0.001. (**B**) Alizarin Red staining composite microscope images from the different groups of conditioned media and the respective control. Each micrograph represents the whole 12-well and consists of serial micrographs as captured by the Tissue FAXS microscope system. *S. oralis* J22 and dual-species biofilm conditioned media elicited considerable abatement of mineralization compared to *A. naeslundii* T14V-J1 and dual-species planktonic groups respectively. Scale bars represent 1 cm. AN, *A. naeslundii* T14V-J1; SO, *S. oralis* J22; DSP, dual-species planktonic conditioned media; DSB, dual-species biofilm conditioned media; control, mineralizing DMEM (mDMEM).

Irrespective of the origin of the conditioned media, these all significantly reduced the normalized (*i.e*. on a per cell level) extracellular calcium deposited by SCAPs compared to non-stimulated mineralizing controls (mDMEM), as assessed by the colorimetric quantification of the AR-S-stained extracellular calcium deposits (P ≤ 0.001). Conditioned media from *S. oralis* J22 planktonic cultures most strongly inhibited mineralization, while conditioned media from *A. naeslundii* T14V-J1 planktonic cultures had the least influence. Interestingly, conditioned media from the dual-species biofilm (co-cultured *A. naeslundii* and *S. oralis*) had a stronger impact on the mineralization reduction of SCAPs compared to their combined planktonic media, with the latter showing an intermediate inhibitory influence on mineralization (Fig. 3A). Representative micrographs of the whole 12-well plates from each group are presented in Fig. 3B **(**Supplementary Tables 8–10**)**.

### Dentinogenic potential is abolished while mineralization- and inflammation- associated gene expression is regulated in a bacterial species and culture-dependent fashion

**Figure 4:**
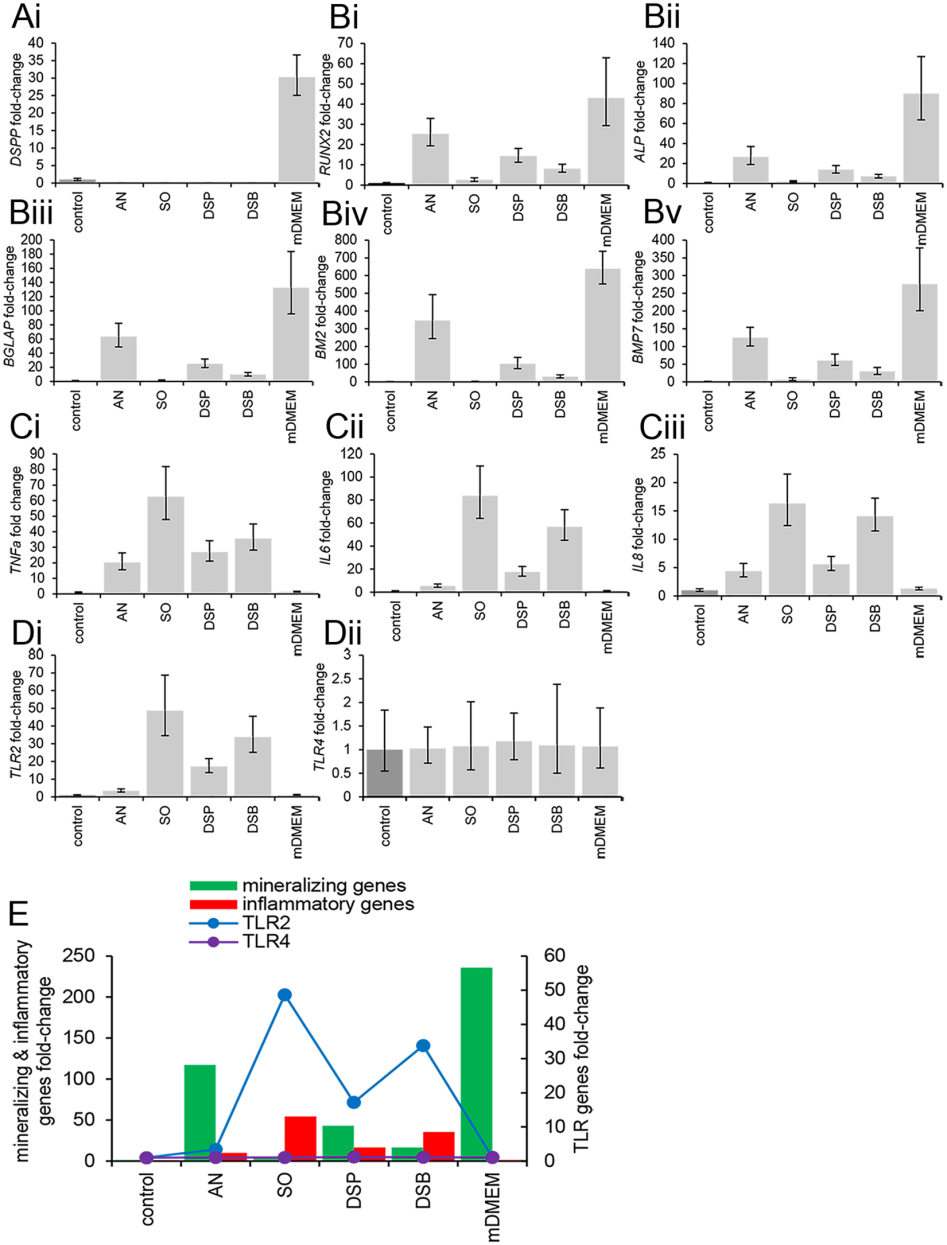
Relative expression of dentinogenic, mineralizing, pro-inflammatory, *TLR2* and *TLR4* genes, as compared to control (DMEM) (fold-change equals to 1) and normalized to B2M reference gene after 14-day exposure of human SCAPs to the different mineralizing conditioned media. (**Ai**) DSPP expression was inhibited and (**Bi–v**) mineralizing genes were significantly downregulated. (**Ci–iii**) Pro-inflammatory genes and (**Di**) *TLR2* showed significantly increased expression, as opposed to the expression of *TLR4* which remained low and unaffected by the exposure to the different conditioned media (**Dii**). Bars represent mean values; error bars indicate Standard Deviation (SD). One-way *ANOVA* tests yielded significant values for all genes (P ≤ 0.001), except for *TLR4*. All pairwise comparisons (*post-hoc Tukey HSD* test) are provided in Supplementary Table 2. (**E**) The gene expression profile chart highlights the overall gene expression pattern of the studied genes, with the pro-inflammatory and *TLR2* genes demonstrating the same trend, opposite to the trend of the mineralizing genes. The expression of *TLR4* remained unaffected by the exposure of SCAPs to the different conditioned media. control, culture medium (DMEM); AN, *A. naeslundii* T14V-J1; SO, *S. oralis* J22; DSP, dual-species planktonic conditioned media; DSB, dual-species biofilm conditioned media; mDMEM, mineralizing DMEM.

Irrespective of the bacterial species or (co)culture conditions, their products abolished expression of *DSPP* in SCAPs during mineralization, while the control showed upregulated expression of *DSPP* (Fig. 4Ai). All assessed relevant mineralization genes (*RUNX2, ALPL, BGLAP, BMP2* and *BMP7*) were downregulated by bacterial products in mineralizing SCAPs (Fig. 4Bi–v). Similar to the influence of bacteria on calcium deposition, conditioned media from planktonic *S. oralis* J22 most strongly inhibited expression of mineralizing genes, while *A. naeslundii* T14V-J1 conditioned media showed only little suppression. In fact, the presence of *A. naeslundii* in planktonic or biofilm co-cultures with *S. oralis* J22 suppressed the inhibitory influence of *S. oralis* on expression of mineralization genes (Fig. 4Bi–v).

Compared to the expression of mineralization genes, secreted products of bacteria had an inverse influence on pro-inflammatory gene expression (*TNFA, IL6* and *IL8*). While controls showed virtually no expression of pro-inflammatory genes, these was strongly upregulated by planktonic *S. oralis* conditioned medium in mineralizing SCAPs, even when *S. oralis* was in biofilm or planktonic co-culture with *A. naeslundii* T14V-J1 (Fig. 4Ci–iii). *A. naeslundii* conditioned medium upregulated all three pro-inflammatory genes too, but to a lesser extent. The strong stimulation of pro-inflammatory gene expression from *S. oralis* was only marginally influenced by *A. naeslundii*, because conditioned media from co-cultures (planktonic and biofilm) still strongly promoted expression of *TNFA, IL6* and *IL8* (Fig. 4Ci–iii).

### *TLR2* (but not *TLR4*) gene expression is regulated in a bacterial species and culture-dependent fashion

The expression of *TLR2*, which is a specific receptor for Gram^+^ pathogen-associated molecular patterns (PAMPs), *i.e*. bacterial products, was low on mineralizing SCAPs (Fig. 4Di). Incubation with conditioned media upregulated *TLR2* expression, irrespective of source or culture condition. The expression pattern, however, was remarkably similar to the expression of pro-inflammatory genes (Fig. 4Ci–iii, Di), with a relatively low upregulation induced by *A. naeslundii* T14V-J1 conditioned medium and high upregulation by conditioned media from *S. oralis* monocultures or co-cultures with *A. naeslundii* T14V-J1, either planktonic or in biofilm (Fig. 4Di). The expression of TLR4 remained unaltered and remarkably low after incubation of SCAPs either with the control mineralizing media (MDMEM) or with the various conditioned media (Fig. 4Dii).

Overall, a correlative gene expression profile chart highlighted the gene expression trend among the groups and justified the hypothesis that the abatement of the mineralization efficiency of human SCAPs could be mediated primarily through the TLR2 signaling transduction pathway (Fig. 4E).

Detailed pairwise comparisons and statistically significant differences are presented in Supplementary Table 13.

### Bacterial activation of TLR signaling inhibits mineralization

**Figure 5:**
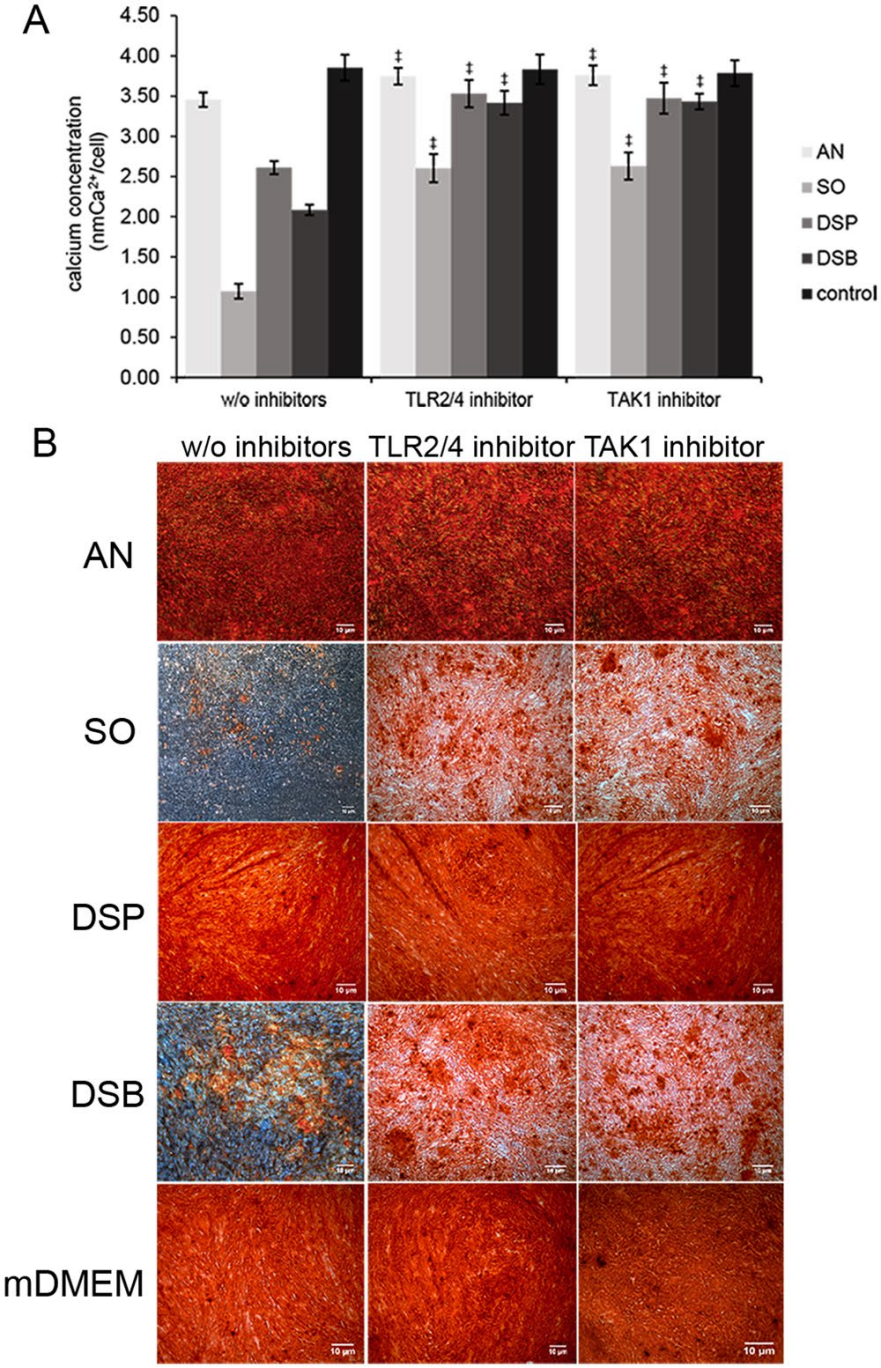
Mineralization capacity of human SCAPs 14 days after continuous blockade of TLR2/4 or TAK1 and exposure to the different mineralizing conditioned media. **(A)** Comparison of the extracellular calcium deposited by human SCAPs (normalized per cell level) exposed to the TLR2/4- and TAK1- supplemented mineralizing conditioned media against the un-supplemented mineralizing control media (independent samples *t-test*). Significantly increased deposition of extracellular calcium was detected in all groups after blockade of the signaling (^‡^ for P ≤ 0.001), except from the control groups (mDMEM). Bars represent mean values; error bars indicate Standard Deviation (SD). (**B**) Alizarin Red staining microscope images demonstrating the re-establishment of SCAPs mineralization efficiency after blocking upstream (TLR2/4 inhibitor) and downstream (TAK1 inhibitor) the TLR pathway. Scale bars represent 10 μm. w/o inhibitors, without inhibitors; AN, *A. naeslundii* T14V-J1; SO, *S. oralis* J22; DSP, dual-species planktonic; DSB, dual-species biofilm; control, mineralizing DMEM (mDMEM).

To corroborate the hypothesis of TLR involvement in the attenuation of the mineralization efficiency of human SCAPs, AR-S staining was performed after the continuous pharmacological inhibition of the TLR signaling, either after adding a TLR2/4 inhibitor (upstream) or a TAK1 inhibitor (downstream) during the 14-day exposure of the cells to the various bacterial conditioned media. Quantification of the extracted dye was performed, as described previously. No difference was observed in the mineralizing control groups (mDMEM) after TLR2/4 or TAK1 blockade. All experimental groups showed a significant increase in terms of extracellular amounts of deposited calcium, after prolonged upstream TLR2/4 blockade or downstream TAK1 blockade (P ≤ 0.001) (Fig. 5A). Upon visualization, the density of the stained calcium deposits had increased in all experimental groups after both upstream (TLR2/4) and downstream (TAK1) blocking of TLR signaling, compared to the corresponding controls groups without inhibitors. Microscopically, this difference was conspicuously visible in the groups exposed to the conditioned media derived from *S. oralis* J22 and dual-species biofilm cultures, in which mineralization was also markedly decreased before any inhibition had taken place (Fig. 5B).

## Discussion

The main finding of our study was that exposure to products from endodontopathogenic bacteria (*S. oralis* J22 and *A. naeslundii* T14V-J1) inhibited mineralization of human SCAPs in a TLR-dependent fashion. This was evidenced by the re-establishment of SCAPs mineralization capacity firstly, after blocking upstream the TLR signaling pathway and secondly after blocking downstream one of the pivotal mediators of TLR activation (and also a central hub of inflammatory signaling-TAK1-). Evidence at the gene expression level pointed out clearly towards the TLR2 (and not TLR4) as being the pivotal molecule regulating this effect. A second finding was that the degree of inhibition of mineralization was highly dependent on the bacterial species and their growth mode (biofilm *vs*. planktonic cultures). *S. oralis* J22, grown either as planktonic monoculture or in dual-species biofilms with *A. naeslundii* T14V-J1, strongly inhibited mineralization and caused a significant upregulation of inflammatory markers in SCAPs. *A. naeslundii* T14V-J1 induced only a low degree upregulation of SCAPs inflammatory markers, which was coupled with a lower inhibition of mineralization as well. Allegedly, the bacterial products of *A. naeslundii* T14V-J1 appeared to rescue the inhibition of mineralization induced by *S. oralis* J22 products, while mitigating also the inflammatory phenotypical shift of SCAPs. However, this rescuing effect seemed to be highly dependent on the growth mode, as bacterial products of dual-species biofilms compromised mineralization capacity of SCAPs stronger than products from their respective mixed planktonic cultures.

The exposure of SCAPs to planktonically and biofilm-released bacterial products led to a different degree of upregulated *TLR2* gene expression. A well-established fact is that specific bacterial structures derived from Gram-positive bacteria elicit TLR2-mediated responses from eukaryotic cells equipped with this recognition molecule^37^. However, this is the first report that demonstrates different TLR2-dependent dental stem cell responses after exposure to different Gram-positive species. This call for studies that investigate TLR-mediated dental stem cell behavior on a species-dependent and not only on specific bacterial structural motif level. *S. oralis* and *A. naeslundii* are clinically relevant bacterial species; both are resistant to endodontic disinfection procedures and persistently present even in previously disinfected root canals^35,36^. Although their capacity to aggregate and form robust multi-species biofilms is established^43^, the mechanisms of their impact on stem cell fate are poorly investigated. Indeed, exposure to *S. oralis* products had detrimental effects on the basic cell processes and the mineralizing efficiency of SCAPs. For this specific bacterial species, surface components (other than the frequently reported LTA and peptidoglycans) involved to bacterial adhesion and colonization events might have contributed to this considerably high *TLR2* gene expression and the inflammatory SCAPs polarization observed^44,45^. Interestingly, *A. naeslundii* T14V-J1 products led only to a minor decline. Indeed, when *A. naeslundii* T14V-J1 was combined with *S. oralis* J22, irrespective of the culture or growth conditions, inhibition of mineralization seemed to be dampened. To the best of our knowledge, there is no specific evidence describing the action of A. naeslundii on SCAPs or other dental stem cells, thus making any speculations on the observed responses rather precarious. The componential characterization of the conditioned media could elucidate the intriguing results of this study and further research is warranted. However, taking as a fact that *A.naeslundii T14V-J1* products impair SCAPs mineralization capacity only to a minor degree, a ‘dilution effect’ of the deleterious components derived from the *S. oralis* J22 cannot be excluded. Thus, the rescuing effect of A. naeslundii *T14V-J1* observed could merely reflect a quantitative shift of the conditioned media components towards a more favorable equilibrium for the SCAPs.

Especially for the SCAPs response to the dual-species biofilm conditioned media, components of the biofilm matrix should be acknowledged as a contributing factor. The production of matrix components, such as polysaccharides and proteins vary depending on growth mode (planktonic *vs* biofilm) both in qualitative and quantitative terms, with biofilm outperforming the respective planktonic cultures^46,47^. Also, the presence of biofilm matrix components seems capable of triggering TLR2 signaling^48,49^. Moreover, during the phenotypic switch from planktonic to biofilm phase, the secreted or released bacterial products could change^50,51^. It has been shown that the osteogenic differentiation of human bone marrow mesenchymal stem cells is inhibited by biofilm components in a TLR2-dependent fashion^52^. Our results corroborate these findings, because biofilm-derived products compromised significantly the osteogenic differentiation of SCAPs in a TLR2-dependent fashion.

Osteogenesis and dentinogenesis are governed by similar stimuli and underlying pathways, yet formation of dentin is specific for teeth and requires expression of *DSPP*^53^. Interestingly, it has been reported that residual mono-species *Enterococcus faecalis* biofilms abrogate the dentinogenic potential of SCAPs, while their osteogenic capacity is maintained^22^. Our data concur partly with these results with regard to SCAPs only concerning the dentinogenic potential, as planktonic and biofilm bacterial products led to complete loss of *DSPP* gene expression. However, in our study, products from *S. oralis J22* and *A. naeslundii* T14V-J1 planktonic and biofilm cultures caused a downregulation of the mineralizing gene profile expression, with planktonic *S. oralis* J22 and the dual-species biofilm cultures eliciting a much more considerable effect. This indicates a species-dependent influence on osteogenic plasticity of SCAPs that likely depends on the composition and concentration of bacterial products.

Binding of ligands from oral bacteria or their biofilms, activates TRL2 in TLR2-bearing cells^54,55^ and recruits adaptor protein myeloid-differentiation primary-response protein 88 (MyD88). This activates transforming growth factor-β (TGF-β)-activated kinase (TAK1), a pivotal pro-inflammatory factor, that in turn activates the major inflammatory transcription factors nuclear factor-κB (NF-κB) and p38^56^. Their activation upregulates expression of pro-inflammatory genes such as *IL1B*, *IL6, IL8* and *TNFA*^57,58^. In this study, increased gene expression of *TLR2*, *IL6, IL8* and *TNFA* correlated negatively with the expression of mineralizing genes and was associated with a reduced mineralizing capacity of SCAPs. Long-term exposure of SCAPs to pro-inflammatory stimuli has been shown to inhibit mineralization and reduce the expression of osteogenic and dentinogenic genes^59^. Pro-inflammatory stimulation of human DPSCs with TNFα impairs their mineralizing efficiency, that is rescued through inhibition of NF-κB, a major downstream mediator^60,61^. Our results show that inhibition of pro-inflammatory pathway mediators (TLR2/4 and TAK1) restore the mineralization capacity of SCAPs, which corroborates the findings with the DPSCs. The products of *S. oralis* caused a stronger pro-inflammatory activation than products of *A. naeslundii*, which might relate to differences in composition and concentration of products released by these bacteria. However, it still remains unclear why *A. naeslundii* products appeared to alleviate the pro-inflammatory stimulation by *S. oralis* products.

A pivotal transcription factor in osteogenic differentiation is Runx2, which is activated by mitogen-activated protein kinase (MAPK) p38^62^. Activation of p38 may occur canonically by activated TAK1, which could be activated by TLR2-bound bacterial ligands. TLR-activated p38 activates pro-inflammatory genes such as *IL1B, TNFA, IL8*, and *IL6*. Alternatively, non-canonically activated p38, e.g. by growth factors bound to receptor tyrosine kinases or by Wnt signaling, activates Runx2 that drives osteogenesis subsequently^63^. *In vitro*, MAPKs regulate of osteogenic differentiation of human MSCs^64^. Dental pulp stem cells which are closely related to SCAPs, also depend on growth factor-activated p38 for osteogenic differentiation^65^. Interestingly, ERK1/2 MAPK inhibits osteogenesis, while p38 MAPK promotes osteogenic differentiation of human DPSCs^66^. Our results suggest that bacterial triggering of p38-induced pro-inflammatory activation supersedes growth factor-activated p38-driven osteogenesis. The underlying mechanism is unclear, but may relate to the increased expression of *TLR2* which could amplify the pro-inflammatory signal, while the osteogenic stimulus is unchanged. This causes a fate shift from osteogenesis to inflammation. This is corroborated by the rescue of osteogenesis after inhibition of TLR2 or TAK1.

In summary, this study has highlighted the consequences that biofilm-derived components bear on relevant dental pulp repair processes that are dependent on proper stem cell function and differentiation. Moreover, it has demonstrated the diverse effects that various PAMPs can have on stem cells functions. SCAPs acquired an immunological rather than a mineralizing phenotype when exposed to bacterial stimuli. The overall results suggest that the TLR2 pathway may act as a checkpoint of this immunophenotypical transition. Whether a cross-talk exists between the TLR2-induced pro-inflammatory activation and the signaling related to the mineralization of human SCAPs warrants further investigation. Our findings have clinical relevance and could facilitate the stem cell-mediated endodontic regenerative procedures. Firstly, they highlight the need for improvement of the disinfection protocols that could result in lowering the microbial burden to levels incapable of altering the stem cell functions. Secondly, they point towards the judicious manipulation of endogenously introduced human SCAPs (or exogenously implanted dental stem cells) in order to modify the untoward events of TLR-priming and signaling.

### Data availability

The datasets generated and analysed during the current study are available from the corresponding author on reasonable request.

## Electronic supplementary material

 Supplementary Information
