# Supplementary material for: Secreted products of oral bacteria and biofilms impede mineralization of apical papilla stem cells in TLR-, species-, and culture-dependent fashion
Source: Sci Rep. 2018 Aug 21;8:12529. doi: 10.1038/s41598-018-30658-5 (PMC6104064; doi:10.1038/s41598-018-30658-5)
Supplement: Supplementary file 1 — Supplementary Information [file 41598_2018_30658_MOESM1_ESM.docx]

**Secreted products of oral bacteria and biofilms impede mineralization of apical papilla stem cells in TLR-, species-, and culture-dependent fashion.**

Xenos Petridis^1^*, Luc W. M. van der Sluis^1^, René J.B. Dijkstra^1^, Marja G.L. Brinker^2^, Henny C. van der Mei^3^, Martin C. Harmsen^2^

^1^University of Groningen, University Medical Center Groningen, Center for Dentistry and Oral Hygiene, Groningen, The Netherlands

^2^University of Groningen, University Medical Center Groningen, Department of Pathology and Medical Biology, Groningen, The Netherlands

^3^University of Groningen, University Medical Center Groningen, Department of Biomedical Engineering, Groningen, The Netherlands

*Corresponding author:

Xenos Petridis, DDS, MSc in Endodontics, e-mail: x.petridis@umcg.nl.

Address: Antonius Deusinglaan 1, 9713 AV Groningen, P.O Box 196, 9700 AD Groningen, The Netherlands

**Supplementary Methods S1**

1x10^6^ SCAP were suspended in FACS buffer consisting of Dulbecco’s phosphate buffered saline (Ca^++^, Mg^++^ free) (Lonza Biowhittaker, Verviers, Belgium) and 1% (w/v) bovine serum albumin (Sigma-Aldrich, Boston, MA, USA) (PBS/1% BSA). Next, the cells were incubated for 30 min at 4˚C in the dark with the following anti-human monoclonal antibodies: CD31-phycoerythrine/cyanine7 (Pe/Cy7; IQ Products, Groningen, The Netherlands), CD45-fluorescein isothiocyanate (FITC; IQ Products), CD90- allophycocyanin (APC; BD Bioscience, San Jose, CA, USA), CD29-APC (eBiosience, Vienna, Austria), CD44 FITC (BD Bioscience) and CD105-Pe/Cy7 (eBioscience). As isotype controls, the following monoclonal antibodies were used: mouse IgG1 kappa-Pe/Cy7 (eBioscience), mouse IgG1 kappa-APC (eBioscience) and mouse IgG1 kappa-FITC (Biolegend, San Diego, CA, USA). Cells were washed three times with FACS buffer and after the last centrifugation (300xg, 5 min, 4^0^C) they were suspended in 200 μL FACS buffer before FACS analysis.

**Supplementary Methods S2**

1x10^4^ SCAP/well were seeded in 24-well plates (Corning® Costar®, Sigma-Aldrich) and incubated with culture medium. Upon reaching confluency, the medium was replaced by osteogenic, adipogenic or smooth muscle cell differentiation medium. Osteogenic medium consisted of culture medium supplemented with 100 nM dexamethasone, 10 mM b-glycerophosphate and 50 μM ascorbic acid. Adipogenic medium consisted of culture medium supplemented with 100 nM dexamethasone, 1 nM insulin, 0.5 mM isobutymethylxanthine. Smooth muscle cell differentiation medium consisted of culture medium with 10 ng/mL Transforming Growth factor beta-1 (TGF-β1) (PeproTech, London). Cells were maintained in the differentiation media for 14 days with refreshment every 3 days. After 14 days, cells were fixed with 2% paraformaldehyde (PFA) in PBS for 30 min and stained with 40mM Alizarin Red-S (AR-S, pH 4.2) (Sigma Aldrich) to show mineralization (osteogenic differentiation), Oil-Red-O (Sigma- Aldrich, St. Louis, MO) to show lipid accumulation (adipogenic differentiation) and Phalloidin-FITC (Invitrogen, Thermo Fisher Scientific), diluted 1:250 in PBS containing 1 µg/mL 4’,6-diamidino-2-phenylindole (DAPI) for 30 min to detect the F-actin cytoskeleton (smooth muscle cell differentiation). AR-S and Oil-Red-O staining were evaluated with an inverted light microscope (Leica Microsystems, DM IL). Phalloidin-FITC staining was evaluated with an inverted fluorescent microscope (Leica Microsystems, DM IL).

**Supplementary Methods S3**

SCAP (passage 3) were seeded onto flat bottom multi-well plates (Corning® Costar® 96-Well Cell Culture Plates, Sigma-Aldrich) at a cell density of 1x10^3^ cells/well and incubated with 150 µL culture medium in a humidified incubator at 37˚C with 5% CO_2_ for 24 h. Next, 150 µL conditioned media serially diluted in culture media was added to each well. At the end-point (3 days), the wells were washed once with 150 µL PBS and 150 µL of culture media containing 0.5 mg/mL MTT (Sigma-Aldrich, Amsterdam, the Netherlands) was added. The plates were incubated for 4h in a humidified incubator at 37˚C with 5% CO_2._ Following incubation, media were decanted, 150 µL of DMSO (Dimethyl Sulfoxide) (Sigma-Aldrich, Amsterdam, the Netherlands) was added to each well and the plates were covered with aluminium foil and agitated on an orbital shaker for 15 min. Next, the absorbance was measured at a wavelength of 570 nm (with a reference filter of 650nm) with a Benchmark microplate reader (Bio-Rad Laboratories, Hercules, CA).

**Supplementary Methods S4**

Following 30 min fixation in 2% PFA in PBS, SCAP were washed with PBS and permeabilized with 0.5% Triton X-100 (Sigma-Aldrich, MO, USA) in PBS for 10 min. Next, they were incubated with 10% goat serum in PBS for 30 min to prevent non-specific binding of primary antibodies. This was followed by incubation at room temperature for 90 min with anti-human Ki-67 rabbit monoclonal antibody (Abcam, the Netherlands), diluted 1:250 in PBS containing 10% donkey serum and 1µg/ml 4’,6-diamidino-2-phenylindole (DAPI). Subsequently, samples were washed with 0.05% Tween-20 in PBS and incubated in dark conditions at room temperature for 30 min with donkey anti-rabbit IgG (H+L) cross-adsorbed Alexa Fluor 594 secondary antibody (Invitrogen), diluted 1:500 in 2% normal human serum in PBS. Thorough washing steps with 0.05% Tween-20 in PBS followed.

**Supplementary Methods S5**

Prior to the initiation of the dye extraction procedures, sterile demineralized water (sdH_2_O) was aspirated, plates were left at an angle to dry and stored at -20˚C for 24 h. Next day, 10% (v/v) acetic acid was added to each well, and the plates were incubated at room temperature for 30 min with gentle shaking. Then, the monolayer was scraped from the plates with a cell scraper and transferred with the acetic acid to microcentrifuge tubes. After vortexing for 30 s, the slurry was heated to 85˚C for 10 min and transferred to ice for 5 min. The supernatants were collected by centrifugal force (20,000xg, 4˚C, 15 min) and transferred to a new microcentrifuge tube. Next, 200 mL of 10% (v/v) ammonium hydroxide was added to restore the pH between 4.1-4.5. Finally, 150 μL aliquots were transferred to a flat bottom 96-well plate and the absorbance was measured at a wavelength of 405 nm with a Benchmark microplate reader. The exact AR-S concentration was calculated from the trendline equation (R^2^ ~ 1) derived from a standard curve made by plotting absorbance (405 nm) as a function of AR-S standard concentrations and normalized to the number of the DAPI stained nuclei for each group.

**Supplementary Methods S6**

RNA aliquots of 1 μg (from the concentrated total RNA extracted from SCAP) were used for M-MuLV and random hexamer primer reverse transcription to obtain complementary DNA (cDNA) with the RevertAid First Strand cDNA synthesis kit (ThermoFisher Scientific) and following the manufacturer’s guidelines. Briefly, cDNA synthesis was carried out in a final 20 μL volume containing template RNA (1 μg), random hexamer primer (12 μg), RiboLock RNase Inhibitor (20U), 1 mM dNTP mix, RevertAid M-MuLV Reverse Transcriptase (200 U), 1X reaction buffer and nuclease-free water. After the addition of the RNA template, the random hexamer primer and the nuclease-free water, the mix was incubated at 65˚C, for 5 min for breaking any secondary structures. Then, the rest of the components were added and the mix was incubated at 25˚C for 5min, followed by an incubation step at 42˚C for 60 min and a reaction termination step at 70˚C for 5 min.

**Supplementary Methods S7**

The RT-qPCR 10 μL reaction contained 4.5 μL cDNA (2 ng/μl) from each sample mixed with 5 μL 2X iQ SYBR Green Supermix (Bio-Rad) and 0.25 μL of each sense and antisense primers (6μM). The real-time qPCR reactions were performed in the Vii A7 Real-Time PCR system (ThermoFisher Scientific) with the following amplification conditions: an initial denaturation step at 95°C for 10 min and then 40 cycles of 15 s at 95˚C, 1 min at 60˚C and 30 s at 72˚C. The specificity of the qPCR products was evaluated by melting curve analysis.

| Gene (human) | Forward primer (5’-3’) | Reverse primer (5’-3’) | NCBI RefSeq |
| --- | --- | --- | --- |
| *DSPP* | GGGAATAGAAATCAAGGGTC | CAAGATCATTCCATGTTGTCC | NM_0142018 |
| *RUNX2* | AAGCTTGATGACTCTAACC | TCTGTAATCGACTCTGTCC | NM_001015051 |
| *ALPL* | TCTTCACATTTGGTGGATAC | ATGGAGACATTCTCTCGTTC | NM_000478 |
| *BGLAP (osteocalcin)* | TTCTTTCCTCTTCCCCTTG | CCTCTTCTGGAGTTTATTTGG | NM_199173 |
| *BMP2* | CGGACTGCGGTCTCCTAA | GGAAGCAGCAACGCTAGAAG | NM_001200 |
| *BMP7* | ACCACTGGGTGGTCAATCC | CAACTTGGGGTTGATGCTCT | NM_001719 |
| *TNFA* | CAGCCTCTTCTCCTTCCTGAT | GCCAGAGGGCTGATTAGAGA | NM_000594 |
| *IL6* | AGCTCAATAAGAAGGGGCCTA | TGAGAAACCCTGGCTTAAGTAGA | NM_000600 |
| *IL8* | CTTTCAGAGACAGCAGAGCA | ACACAGAGCTGCAGAAATCA | NM_000584 |
| *TLR2* | CTTTCAACTGGTAGTTGTGG | GGAATGGAGTTTAAAGATCCTG | NM_003264 |
| *TLR4* | CTGCGTGGAGGTGGTTCCTA | CAGGTCCAGGTTCTTGGTTGAG | NM_003266 |
| *B2M* | TTCTGGCCTGGAGGCTATC | TCAGGAAATTTGACTTTCCATTC | NM_004048 |

**Supplementary Table 1.** Primer sequences used in RT-qPCR analysis.

*DSPP*, dentin sialophosphoprotein; *RUNX2*, Runt-related transcription factor 2; *ALPL*, alkaline phosphatase; *BGLAP*, bone gamma-carboxyglutamic acid-containing protein; *BMP2*, bone morphogenetic protein 2; *BMP7*, bone morphogenetic protein 7; *TNFA*, tumor necrosis factor alpha; *IL6*, interleukin 6; *IL8*, interleukin 8; *TLR2*, Toll-like receptor 2; *B2M*, TLR4, Toll-like receptor 4; β2 microglobulin; NCBI RefSeq, National Center for Biotechnology Information Reference Sequence.

**Supplementary Figure S1 (a-e)**


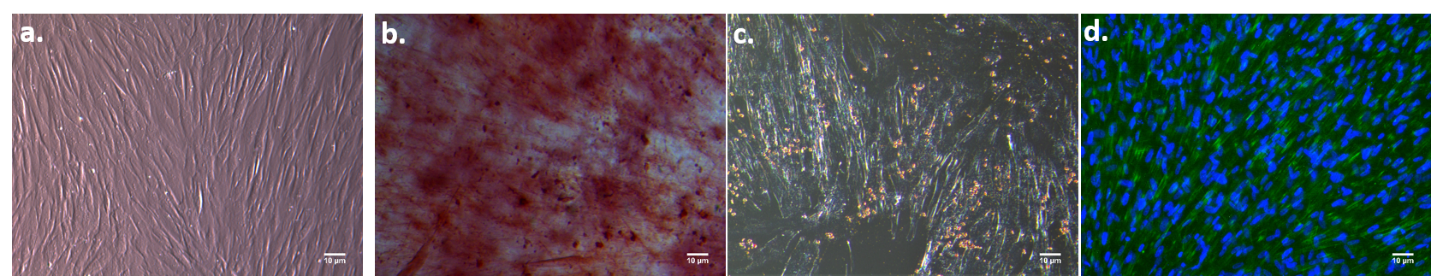
**
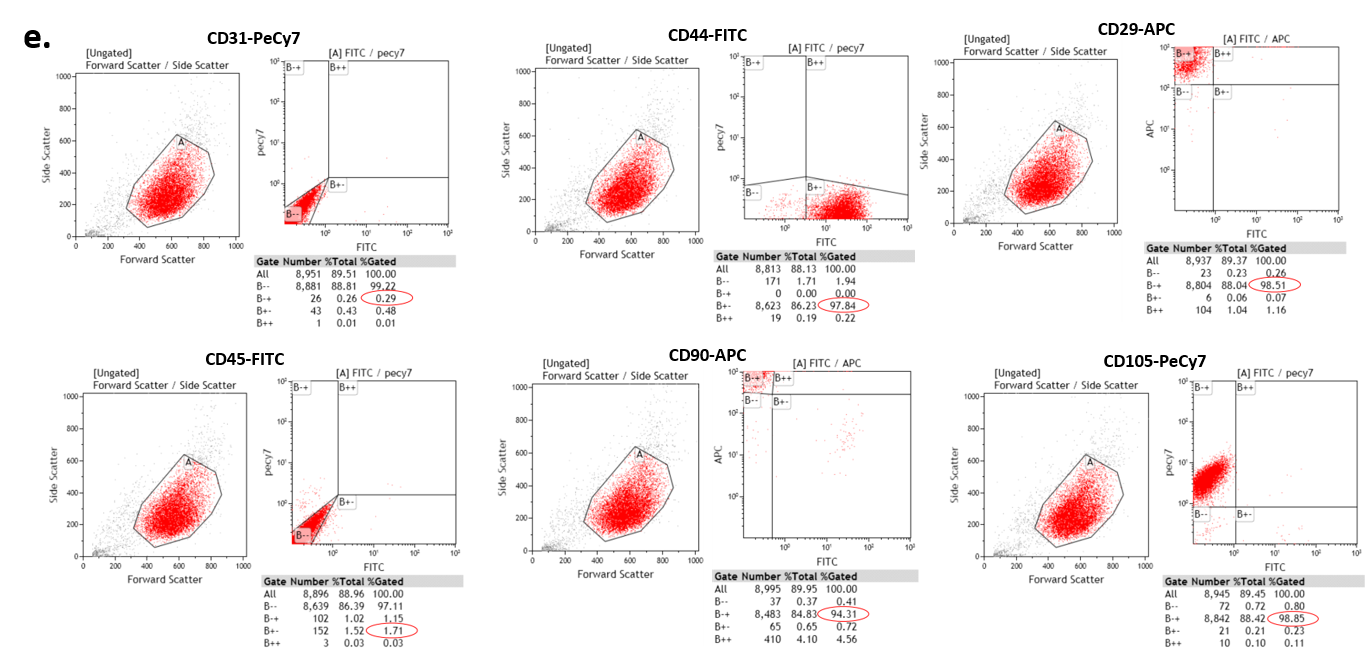
**

Characterization of stem cells from the apical papilla (SCAP) (passage 3). **(a)** Typical fibroblast-like cell morphology (elongated and spindle-shaped cells). **(b)** Osteogenic differentiation of SCAP, calcium deposits stained red, after Alizarin Red staining. **(c)** Adipogenic differentiation of SCAP, lipid droplet accumulation stained red, after Oil-Red-O staining. **(d)** Smooth muscle cell-like differentiation of SCAP, F-actin stained green and nuclei stained blue, after phalloidin-FITC and DAPI staining respectively. **(e)** Flow cytometry analysis showed high positivity for cell surface markers associated with mesenchymal stromal cell phenotypes (CD- 44, 29, 90, 105) and extremely low positivity for markers associated with endothelial (CD31) and hematopoietic (CD45) cells.

**Supplementary Figure S2**

Determination of the optimal maximum concentration of conditioned media with MTT cytotoxicity assay. The semi-logarithmic plot shows the abrupt decline of SCAP mitochondrial activity for all conditioned media, when concentrations exceed the 25% threshold. The optimal maximum concentration was set at 25% (all conditioned media were diluted in DMEM at a ratio of 1:3 respectively). AN, *A. naeslundii* T14V-J1; SO, *S. oralis* J22; DSP, dual-species planktonic; DSB, dual-species biofilm.

| **Supplementary Table 2. Descriptive statistics for MTT assay.** | | | | | | |
| --- | --- | --- | --- | --- | --- | --- |
| **Outcome measure: Optical Density 570nm** | | | | | | |
| Conditioned media | N | Mean | Standard deviation | Standard Error | 95% Confidence Interval for Mean | |
|  |  |  |  |  | Lower Bound | Upper Bound |
| AN(a) | 9 | 3.5833 | .14866 | .04955 | 3.4691 | 3.6976 |
| SO(b) | 9 | 2.0500 | .14405 | .04802 | 1.9393 | 2.1607 |
| DSP(c) | 9 | 3.3200 | .11565 | .03855 | 3.2311 | 3.4089 |
| DSB(d) | 9 | 2.2389 | .24060 | .08020 | 2.0540 | 2.4238 |
| control(e) | 9 | 4.1022 | .20993 | .06998 | 3.9409 | 4.2636 |

| **Supplementary Table 3. *Analysis of Variance* (*ANOVA*) for MTT assay.** | | | | | |
| --- | --- | --- | --- | --- | --- |
| **Outcome measure: Optical Density 570nm** | | | | | |
|  | Sum of Squares | df | Mean Square | F | Sig. |
| Between Groups | 28.098 | 4 | 7.025 | 222.042 | **.000** |
| Within Groups | 1.265 | 40 | .032 |  |  |
| Total | 29.364 | 44 |  |  |  |

| **Supplementary Table 4. Multiple comparisons (*post-hoc* *Tukey HSD* test) for MTT assay.** | | | | | | |
| --- | --- | --- | --- | --- | --- | --- |
| **Outcome measure: Optical Density 570nm** | | | | | | |
| (I) conditioned media | (J) conditioned media | Mean Difference (I-J) | Standard Error | Sig. | 95% Confidence Interval | |
|  |  |  |  |  | Lower Bound | Upper Bound |
| AN(a) | SO(b) | 1.53333^*^ | .08385 | **.000** | 1.2939 | 1.7728 |
|  | DSP(c) | .26333^*^ | .08385 | **.025** | .0239 | .5028 |
|  | DSB(d) | 1.34444^*^ | .08385 | **.000** | 1.1050 | 1.5839 |
|  | control(e) | -.51889^*^ | .08385 | **.000** | -.7584 | -.2794 |
| SO(b) | AN(a) | -1.53333^*^ | .08385 | **.000** | -1.7728 | -1.2939 |
|  | DSP(c) | -1.27000^*^ | .08385 | **.000** | -1.5095 | -1.0305 |
|  | DSB(d) | -.18889 | .08385 | .182 | -.4284 | .0506 |
|  | control(e) | -2.05222^*^ | .08385 | **.000** | -2.2917 | -1.8127 |
| DSP(c) | AN(a) | -.26333^*^ | .08385 | **.025** | -.5028 | -.0239 |
|  | SO(b) | 1.27000^*^ | .08385 | **.000** | 1.0305 | 1.5095 |
|  | DSB(d) | 1.08111^*^ | .08385 | **.000** | .8416 | 1.3206 |
|  | control(e) | -.78222^*^ | .08385 | **.000** | -1.0217 | -.5427 |
| DSB(d) | AN(a) | -1.34444^*^ | .08385 | **.000** | -1.5839 | -1.1050 |
|  | SO(b) | .18889 | .08385 | .182 | -.0506 | .4284 |
|  | DSP(c) | -1.08111^*^ | .08385 | **.000** | -1.3206 | -.8416 |
|  | control(e) | -1.86333^*^ | .08385 | **.000** | -2.1028 | -1.6239 |
| control(e) | AN(a) | .51889^*^ | .08385 | **.000** | .2794 | .7584 |
|  | SO(b) | 2.05222^*^ | .08385 | **.000** | 1.8127 | 2.2917 |
|  | DSP(c) | .78222^*^ | .08385 | **.000** | .5427 | 1.0217 |
|  | DSB(d) | 1.86333^*^ | .08385 | **.000** | 1.6239 | 2.1028 |

*. The mean difference is significant at the 0.05 level.

| **Supplementary Table 5. Descriptive statistics for proliferation assay.** | | | | | | |
| --- | --- | --- | --- | --- | --- | --- |
| **Outcome measure: % Ki-67^+^ / DAPI^+^** | | | | | | |
| Conditioned media | N | Mean | Standard Deviation | Standard Error | 95% Confidence Interval for Mean | |
|  |  |  |  |  | Lower Bound | Upper Bound |
| AN(a) | 9 | 6.6422 | .48028 | .16009 | 6.2730 | 7.0114 |
| SO(b) | 9 | 2.2433 | .40175 | .13392 | 1.9345 | 2.5521 |
| DSP(c) | 9 | 5.8378 | .40947 | .13649 | 5.5230 | 6.1525 |
| DSB(d) | 9 | 4.6167 | .41458 | .13819 | 4.2980 | 4.9353 |
| control(e) | 9 | 7.2622 | .49462 | .16487 | 6.8820 | 7.6424 |

| **Supplementary Table 6. *Analysis of Variance* (*ANOVA*) results for proliferation assay.** | | | | | |
| --- | --- | --- | --- | --- | --- |
| **Outcome measure: % Ki-67^+^ / DAPI^+^** | | | | | |
|  | Sum of Squares | df | Mean Square | F | Sig. |
| Between Groups | 141.742 | 4 | 35.436 | 181.487 | **.000** |
| Within Groups | 7.810 | 40 | .195 |  |  |
| Total | 149.552 | 44 |  |  |  |

| **Supplementary Table 7. Multiple comparisons (*post-hoc Tukey HSD test*) for proliferation assay.** | | | | | | |
| --- | --- | --- | --- | --- | --- | --- |
| **Outcome measure: % Ki-67^+^ / DAPI^+^** | | | | | | |
| (I) conditioned media | (J) conditioned media | Mean Difference (I-J) | Standard Error | Sig. | 95% Confidence Interval | |
|  |  |  |  |  | Lower Bound | Upper Bound |
| AN(a) | SO(b) | 4.39889^*^ | .20830 | **.000** | 3.8040 | 4.9938 |
|  | DSP(c) | .80444^*^ | .20830 | **.003** | .2095 | 1.3994 |
|  | DSB(d) | 2.02556^*^ | .20830 | **.000** | 1.4306 | 2.6205 |
|  | control(e) | -.62000^*^ | .20830 | **.037** | -1.2149 | -.0251 |
| SO(b) | AN(a) | -4.39889^*^ | .20830 | **.000** | -4.9938 | -3.8040 |
|  | DSP(c) | -3.59444^*^ | .20830 | **.000** | -4.1894 | -2.9995 |
|  | DSB(d) | -2.37333^*^ | .20830 | **.000** | -2.9683 | -1.7784 |
|  | control(e) | -5.01889^*^ | .20830 | **.000** | -5.6138 | -4.4240 |
| DSP(c) | AN(a) | -.80444^*^ | .20830 | **.003** | -1.3994 | -.2095 |
|  | SO(b) | 3.59444^*^ | .20830 | **.000** | 2.9995 | 4.1894 |
|  | DSB(d) | 1.22111^*^ | .20830 | **.000** | .6262 | 1.8160 |
|  | control(e) | -1.42444^*^ | .20830 | **.000** | -2.0194 | -.8295 |
| DSB(d) | AN(a) | -2.02556^*^ | .20830 | **.000** | -2.6205 | -1.4306 |
|  | SO(b) | 2.37333^*^ | .20830 | **.000** | 1.7784 | 2.9683 |
|  | DSP(c) | -1.22111^*^ | .20830 | **.000** | -1.8160 | -.6262 |
|  | control(e) | -2.64556^*^ | .20830 | **.000** | -3.2405 | -2.0506 |
| control(e) | AN(a) | .62000^*^ | .20830 | **.037** | .0251 | 1.2149 |
|  | SO(b) | 5.01889^*^ | .20830 | **.000** | 4.4240 | 5.6138 |
|  | DSP(c) | 1.42444^*^ | .20830 | **.000** | .8295 | 2.0194 |
|  | DSB(d) | 2.64556^*^ | .20830 | **.000** | 2.0506 | 3.2405 |

*. The mean difference is significant at the 0.05 level.

| **Supplementary Table 8. Descriptive statistics for mineralization assay (quantification of extracted Alizarin Red staining and conversion to nanomoles calcium per cell).** | | | | | | |
| --- | --- | --- | --- | --- | --- | --- |
| **Outcome measure: nmCa^2+^ / cell*** | | | | | | |
| Conditioned media | N | Mean | Standard Deviation | Standard Error | 95% Confidence Interval for Mean | |
|  |  |  |  |  | Lower Bound | Upper Bound |
| AN(a) | 9 | 3.4567 | .08718 | .02906 | 3.3897 | 3.5237 |
| SO(b) | 9 | 1.0722 | .09066 | .03022 | 1.0025 | 1.1419 |
| DSP(c) | 9 | 2.6122 | .08028 | .02676 | 2.5505 | 2.6739 |
| DSB(d) | 9 | 2.1000 | .08124 | .02708 | 2.0376 | 2.1624 |
| control(e) | 9 | 3.8533 | .16155 | .05385 | 3.7292 | 3.9775 |

| **Supplementary Table 9. *Analysis of Variance* (*ANOVA*) results for mineralization assay (quantification of extracted Alizarin Red staining and conversion to nanomoles calcium per cell).** | | | | | |
| --- | --- | --- | --- | --- | --- |
| **Outcome measure: nmCa^2+^ / cell*** | | | | | |
|  | Sum of Squares | df | Mean Square | F | Sig. |
| Between Groups | 43.985 | 4 | 10.996 | 1000.310 | **.000** |
| Within Groups | .440 | 40 | .011 |  |  |
| Total | 44.424 | 44 |  |  |  |

| **Supplementary Table 10. Multiple comparisons (*post-hoc Tukey HSD test*) for mineralization assay (quantification of extracted Alizarin Red staining and conversion to nanomoles calcium per cell).** | | | | | | |
| --- | --- | --- | --- | --- | --- | --- |
| **Outcome measure: nmCa^2+^ / cell*** | | | | | | |
| (I) conditioned media | (J) conditioned media | Mean Difference (I-J) | Standard Error | Sig. | 95% Confidence Interval | |
|  |  |  |  |  | Lower Bound | Upper Bound |
| AN(a) | SO(b) | 2.38444* | .04943 | **.000** | 2.2433 | 2.5256 |
|  | DSP(c) | .84444* | .04943 | **.000** | .7033 | .9856 |
|  | DSB(d) | 1.35667* | .04943 | **.000** | 1.2155 | 1.4978 |
|  | control(e) | -.39667* | .04943 | **.000** | -.5378 | -.2555 |
| SO(b) | AN(a) | -2.38444* | .04943 | **.000** | -2.5256 | -2.2433 |
|  | DSP(c) | -1.54000* | .04943 | **.000** | -1.6812 | -1.3988 |
|  | DSB(d) | -1.02778* | .04943 | **.000** | -1.1689 | -.8866 |
|  | control(e) | -2.78111* | .04943 | **.000** | -2.9223 | -2.6399 |
| DSP(c) | AN(a) | -.84444* | .04943 | **.000** | -.9856 | -.7033 |
|  | SO(b) | 1.54000* | .04943 | **.000** | 1.3988 | 1.6812 |
|  | DSB(d) | .51222* | .04943 | **.000** | .3711 | .6534 |
|  | control(e) | -1.24111* | .04943 | **.000** | -1.3823 | -1.0999 |
| DSB(d) | AN(a) | -1.35667* | .04943 | **.000** | -1.4978 | -1.2155 |
|  | SO(b) | 1.02778* | .04943 | **.000** | .8866 | 1.1689 |
|  | DSP(c) | -.51222* | .04943 | **.000** | -.6534 | -.3711 |
|  | control(e) | -1.75333* | .04943 | **.000** | -1.8945 | -1.6122 |
| control(e) | AN(a) | .39667* | .04943 | **.000** | .2555 | .5378 |
|  | SO(b) | 2.78111* | .04943 | **.000** | 2.6399 | 2.9223 |
|  | DSP(c) | 1.24111* | .04943 | **.000** | 1.0999 | 1.3823 |
|  | DSB(d) | 1.75333* | .04943 | **.000** | 1.6122 | 1.8945 |

*. The mean difference is significant at the 0.05 level.

| **Supplementary Table 11. Descriptive statistics for mineralization assay without and with the TLR 2/4 and TAK-1 inhibitors (quantification of extracted Alizarin Red staining and conversion to nanomoles calcium per cell).** | | | | | | |
| --- | --- | --- | --- | --- | --- | --- |
| **Outcome measure: nmCa^2+^ / cell** | | | | | | |
| Conditioned media | N | Mean | Standard Deviation | Standard Error | 95% Confidence Interval for Mean | |
|  |  |  |  |  | Lower Bound | Upper Bound |
| AN w/o inhibitors | 9 | 3.4567 | .08718 | .02906 | 3.3897 | 3.5237 |
| AN +TLR2/4 inhibitor | 9 | 3.7478 | .10497 | .03499 | 3.6671 | 3.8285 |
| AN +TAK1 inhibitor | 9 | 3.7600 | .12278 | .04093 | 3.6656 | 3.8544 |
| SO w/o inhibitors | 9 | 1.0722 | .09066 | .03022 | 1.0025 | 1.1419 |
| SO +TLR2/4 inhibitor | 9 | 2.6044 | .17579 | .05860 | 2.4693 | 2.7396 |
| SO +TAK1 inhibitor | 9 | 2.6289 | .16952 | .05651 | 2.4986 | 2.7592 |
| DSP w/o inhibitors | 9 | 2.6122 | .08028 | .02676 | 2.5505 | 2.6739 |
| DSP +TLR2/4 inhibitor | 9 | 3.5322 | .17028 | .05676 | 3.4013 | 3.6631 |
| DSP +TAK1 inhibitor | 9 | 3.4756 | .19333 | .06444 | 3.3269 | 3.6242 |
| DSB w/o inhibitors | 9 | 2.1000 | .08124 | .02708 | 2.0376 | 2.1624 |
| DSB +TLR2/4 inhibitor | 9 | 3.4178 | .14906 | .04969 | 3.3032 | 3.5324 |
| DSB +TAK1 inhibitor | 9 | 3.4333 | .09592 | .03197 | 3.3596 | 3.5071 |
| control w/o inhibitors | 9 | 3.8533 | .16155 | .05385 | 3.7292 | 3.9775 |
| control +TLR2/4 inhibitor | 9 | 3.8333 | .18426 | .06142 | 3.6917 | 3.9750 |
| control +TAK1 inhibitor | 9 | 3.7856 | .15828 | .05276 | 3.6639 | 3.9072 |

| **Supplementary Table 12. Independent unpaired Student’s t-test results for mineralization assay without and with the TLR 2/4 and TAK-1 inhibitors (quantification of extracted Alizarin Red staining and conversion to nanomoles calcium per cell).** | | | | | | | |
| --- | --- | --- | --- | --- | --- | --- | --- |
|  | | | | | | |  |
| **Outcome measure: nmCa^2+^ / cell*** | | | | | | |  |
| (I) conditioned media | (J) conditioned media | Mean Difference (I-J) | Standard Error Difference | Sig. | 95% Confidence Interval | |  |
|  |  |  |  |  | Lower Bound | Upper Bound |  |
| AN w/o inhibitors | AN  +TLR2/4 inhibitor | -.29111* | .04548 | **.000** | -.38753 | -.19469 |  |
|  | AN  +TAK1 inhibitor | -.30333* | .05019 | **.000** | -.40974 | -.19693 |  |
| SO w/o inhibitors | SO  +TLR2/4 inhibitor | -1.53222* | .06593 | **.000** | -1.67199 | -1.39245 |  |
|  | SO  +TAK1 inhibitor | -1.55667* | .06408 | **.000** | -1.69251 | -1.42082 |  |
| DSP w/o inhibitors | DSP  +TLR2/4 inhibitor | -.92000* | .06275 | **.000** | -1.05303 | -.78697 |  |
|  | DSP  +TAK1 inhibitor | -.86333* | .06978 | **.000** | -1.01126 | -.71541 |  |
| DSB w/o inhibitors | DSB  +TLR2/4 inhibitor | -1.31778* | .05659 | **.000** | -1.43774 | -1.19782 |  |
|  | DSB  +TAK1 inhibitor | -1.33333* | .04190 | **.000** | -1.42216 | -1.24451 |  |
| control w/o inhibitors | control +TLR2/4 inhibitor | .02000 | .08168 | .810 | -.15316 | .19316 |  |
|  | control +TAK1 inhibitor | .06778 | .07539 | .382 | -.09204 | .22760 |  |

*. The mean difference is significant at the 0.05 level.

**Supplementary Table 13.** Pairwise comparisons (Tukey’s post-hoc test) and statistical significance for all genes used in RT-qPCR analysis.

|  | AN | SO | DSP | DSB | mDMEM |
| --- | --- | --- | --- | --- | --- |
| AN | - | ¶^***^, #^***^, $^***^, †^***^, ‡^***^, ¢^***^, ¤^***^, ¥^***^, §^***^ | ¶^*^, #^*^, $^**^, †^***^, ‡^*^, ¤^***^, §^***^ | ¶^***^, #^***^, $^***^, †^***^, ‡^***^, ¢^**^, ¤^***^, ¥^***^, §^***^ | #^***^, $^*^, ‡^**^, ¢^***^, ¤^***^, ¥^***^, §^***^ |
| SO | ¶^***^, #^***^, $^***^, †^***^, ‡^***^, ¢^***^, ¤^***^, ¥^***^, §^***^ | - | ¶^***^, #^***^, $^***^, †^***^, ‡^***^, ¢^***^, ¤^***^, ¥^***^, §^***^ | ¶^***^, #^***^, $^***^, †^***^, ‡^***^, ¢^**^ | ¶^***^, #^***^, $^***^, †^***^, ‡^***^, ¢^***^, ¤^***^, ¥^***^, §^***^ |
| DSP | ¶^*^, #^*^, $^**^, †^***^, ‡^*^, ¤^***^, §^***^ | ¶^***^, #^***^, $^***^, †^***^, ‡^***^, ¢^***^, ¤^***^, ¥^***^, §^***^ | - | ¶^*^, #^*^, $^**^, †^***^, ‡^*^, ¤^***^, ¥^***^, §^**^ | ¶^***^, #^***^, $^***^, †^***^, ‡^***^, ¢^***^, ¤^***^, ¥^***^, §^***^ |
| DSB | ¶^***^, #^***^, $^***^, †^***^, ‡^***^, ¢^**^, ¤^***^, ¥^***^, §^***^ | ¶^***^, #^***^, $^***^, †^***^, ‡^***^, ¢^**^ | ¶^*^, #^*^, $^**^, †^***^, ‡^*^, ¤^***^, ¥^***^, §^**^ | - | ¶^***^, #^***^, $^***^, †^***^, ‡^***^, ¢^***^, ¤^***^, ¥^***^, §^***^ |
| mDMEM | #^***^, $^*^, ‡^**^, ¢^***^, ¤^***^, ¥^***^, §^***^ | ¶^***^, #^***^, $^***^, †^***^, ‡^***^, ¢^***^, ¤^***^, ¥^***^, §^***^ | ¶^***^, #^***^, $^***^, †^***^, ‡^***^, ¢^***^, ¤^***^, ¥^***^, §^***^ | ¶^***^, #^***^, $^***^, †^***^, ‡^***^, ¢^***^, ¤^***^, ¥^***^, §^***^ | - |

AN: *A. naeslundii* T14V-J1, SO: *S. oralis* J22, DSP: dual-species planktonic, DSB: dual-species biofilm, mDMEM: mineralizing DMEM

¶, *RUNX2*; #, *ALPL*; $, *BGLAP*; †, *BMP2*; ‡, *BMP7*; Ȼ, *TNFA*; ¤, *IL6*; ¥, *IL8*; §, *TLR2*

*, p≤0.05; **, p≤0.01; ***, p≤0.001
